# Biosensors: Electrochemical Devices—General Concepts and Performance

**DOI:** 10.3390/bios13010044

**Published:** 2022-12-28

**Authors:** Oleh Smutok, Evgeny Katz

**Affiliations:** Department of Chemistry and Biomolecular Science, Clarkson University, Potsdam, NY 13699-5810, USA

**Keywords:** enzyme-based biosensors, electrochemical biosensors, electron-transfer mediators, enzyme immobilization, signal transducers

## Abstract

This review provides a general overview of different biosensors, mostly concentrating on electrochemical analytical devices, while briefly explaining general approaches to various kinds of biosensors, their construction and performance. A discussion on how all required components of biosensors are brought together to perform analytical work is offered. Different signal-transducing mechanisms are discussed, particularly addressing the immobilization of biomolecular components in the vicinity of a transducer interface and their functional integration with electronic devices. The review is mostly addressing general concepts of the biosensing processes rather than specific modern achievements in the area.

## 1. Introduction

**Figure 1:**
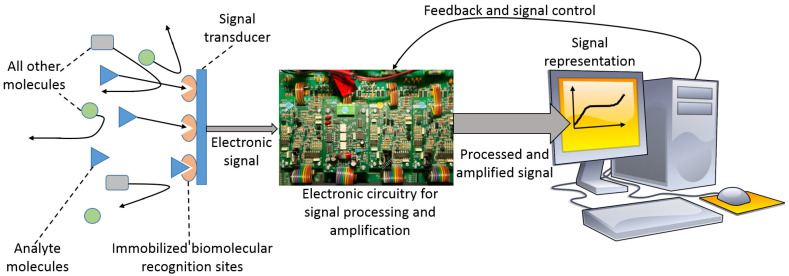
Schematic presentation of a biosensor. The analyte (bio)molecules can be selectively analyzed in a complex mixture containing many other molecules (different molecules are shown schematically with different shapes). The specificity in the analyte detection is provided by biomolecular species immobilized at the signal transducer interface. The electronic component of the biosensor provides the conversion of a chemical signal to an electronic signal with its amplification. The larger size of the second arrow shows an amplified signal. The computer, used in modern devices, allows convenient presentation of the output signal and its processing as needed for specific applications. Notably, the computer is usually miniaturized and integrated with the electronic signal processing part, not being a desktop device, as shown in the scheme for simplicity.

Bioanalytical processes, particularly used in biomolecule assays, are usually based on reactions in solutions between a sample and added various mixtures of reagents, possibly with separation steps following the reactions, then examining the chemical products with optical or electrochemical techniques [1]. On the other hand, biosensors are based on “reagentless” systems, where the needed reagents are integrated components of the systems, and they are not to be added to the reaction solutions, which is an important advantage of the biosensor-based analysis [2]. Another important feature of the biosensor operation is the absence of any special sample pretreatment, which is typical for the great majority of biosensors. These features of biosensors are possible due to the immobilization of all needed reaction components at a transducer interface, then performing the bio/chemical reactions within immobilized layers, followed by transducing the produced chemical reactants to an electronic signal by the transducer device. In other words, the immobilized biomolecule system provides a specific response to the analyte molecules present in a sample, while the physical device converts the chemical product to an amplified electronic signal (Figure 1). The nature of the used transducer and the mechanism of the signal translation depend on the kind of the (bio)chemical process in the immobilized layer and on the biosensor application. Notably, the same (bio)chemical reaction responding to the analyte presence can be technically integrated with various physical transduction methods, e.g., optical [3] or electrochemical [4] transduction methods can be used, and the choice may be based on convenience and required sensitivity. Some similarities can be found between bioassays performed in solutions and biosensor applications, then being different mostly by organizing the reactants in a liquid phase or in an immobilized layer. However, the similarity is not always possible, and some biosensors operate in a way that does not have similar analogs in bioassay processes. For example, biosensors based on field-effect transistors [5] or surface plasmon resonance [6] devices do not have bioassay analogs with the reactions proceeding in solutions. In other words, not all biosensors have analogs in traditional analytical methods, being developed specifically for biosensor applications.

**Figure 2:**
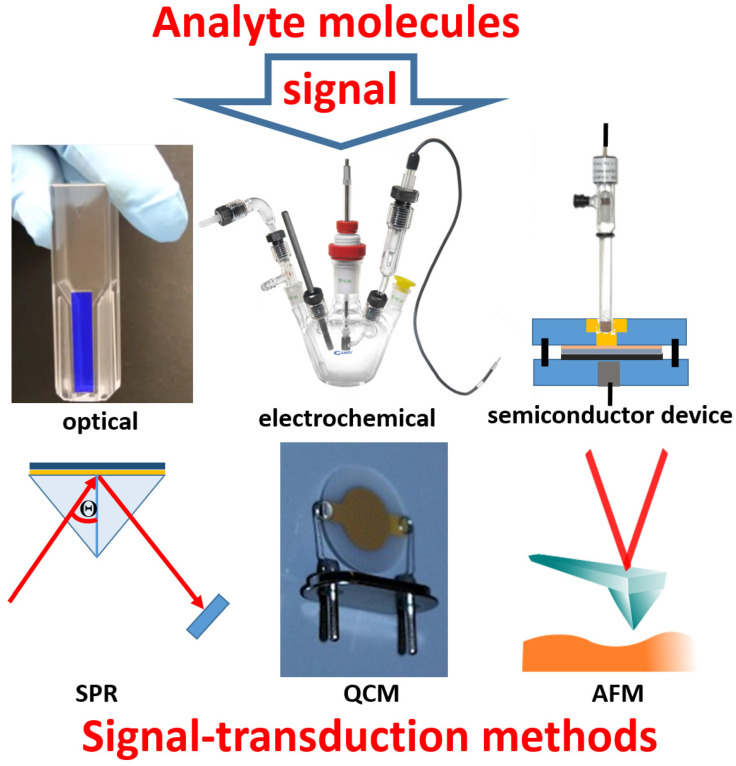
Transduction methods for converting analyte molecule signals to an electronic output: (*i*) optical measurements based on absorbance or fluorescence spectroscopy, (*ii*) various electrochemical measurements including amperometry, potentiometry, impedance spectroscopy, etc., (*iii*) semiconductor measurements, e.g., field-effect transistor signal transduction, using current, potential or capacitance measurements, (*iv*) surface plasmon resonance (SPR) measurements, (*v*) microgravimetric measurements using quartz crystal microbalance (QCM), and (*vi*) nanotechnological methods, e.g., atomic force microscope (AFM) measurements.

It should be noted that many bioanalytical methods performed in solutions might be converted to respective biosensors, but this may not be practical to do. In other words, the technical simplicity and applicability may limit the realization of biosensors with a limited choice of molecule immobilization techniques and signal transduction methods, which are collected in Figure 2. The present review article will mostly overview biosensors based on electrochemical signal transduction methods, leaving many other biosensors to special reviews, which can be found elsewhere. 

## 2. The Immobilized Biomolecule Recognition Element and Physical Signal Transducer

**Figure 3:**
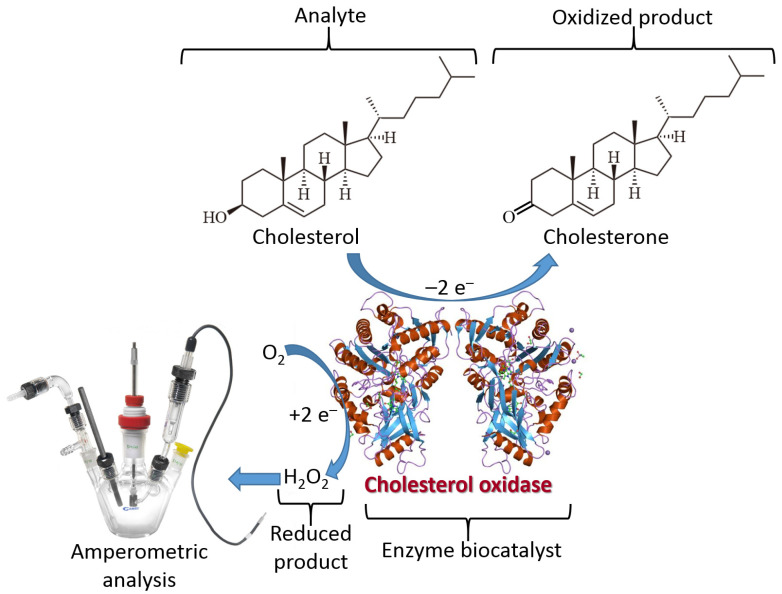
Enzyme-catalyzed reactions can be used for the transformation of a substrate-analyte signal into an electronic signal. The scheme shows an example of such enzyme-based signal transduction using cholesterol oxidase for the analysis of cholesterol. A side H_2_O_2_ product is detected electrochemically, e.g., by amperometric technique. While the measurements are based on the analysis of H_2_O_2_, its concentration is stoichiometrically related to the cholesterol concentration in the analyzed sample. Notably, another approach can be based on an analysis of the O_2_ depletion instead of the H_2_O_2_ production.

**Figure 4:**
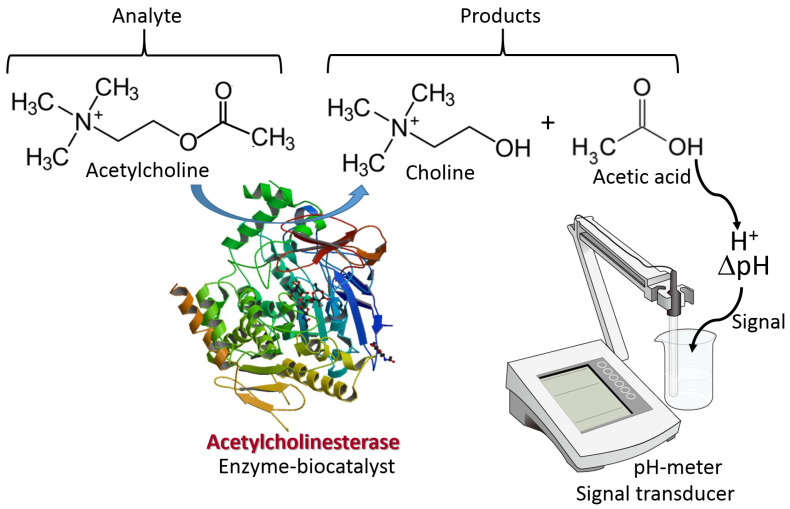
The biomolecule analyte signal biocatalytically converted to pH change, then analyzed with a pH meter operating as a signal transducer. The scheme shows the conversion of acetylcholine analyte to choline and acetic acid catalyzed by acetylcholinesterase enzyme. Biocatalytic signal processing is an example of a general biosensor approach based on pH changes related to the analyte concentration (note that the process should be performed without a buffer solution to allow the pH change). Numerous biosensors based on the same approach have been constructed using the coupling of biocatalytic reactions with measured pH changes. The conventional pH-sensitive glass electrode is shown for simplicity. In real biosensors, miniaturized pH sensors are used. In practical applications, the acetylcholinesterase enzyme inhibitors are analyzed instead of the substrate analysis.

The biomolecule recognition element can be represented by a protein/enzyme, biomacromolecule (e.g., DNA) or other molecule species with affinity to a specific analyte. This element provides a selective response to the analyte molecules and interacts with a transducer surface to allow signal transduction from produced molecule species to an electronic signal. The biomolecule recognition element converts catalytically the analyte molecules to the product species or creates a molecule complex with the analyte. The type of the analyte-recognition element reaction determines the kind of signal transduction and the type of transducer used. While the reaction type and transducer used can be different, two simple examples are shown in Figure 3 and Figure 4 for illustration. Both examples include reactions catalyzed by enzymes, while one results in the production of redox species (i.e., H_2_O_2_), and another produces a pH change (an increase of acidity). The resulting interfacial changes can be analyzed electrochemically using amperometry performed at an enzyme-modified electrode (Figure 3) [7] and pH measurements at an enzyme-modified pH-sensitive glass electrode (Figure 4) [8]. The shown examples were selected for illustration only, not pretending to show the broad variety of possible reactions. Keeping the discussion in the area of enzyme-catalyzed reactions, one can consider many other reaction options, including hydrolysis, esterification, cleavage, etc., while the analyte is an enzyme substrate or enzyme inhibitor.

**Figure 5:**
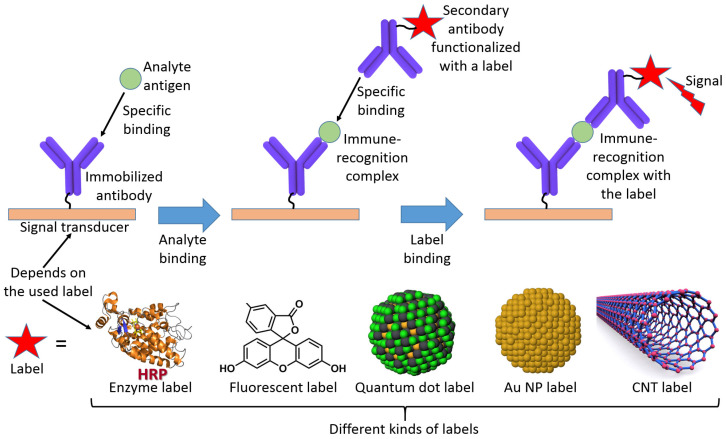
The scheme illustrates a “sandwich”-type immunoassay applied for the analysis of an antigen analyte. The primary antibody operating as a biorecognition species is immobilized at the transducer interface. When an antigen analyte appears in a solution, it produces an affinity antibody–antigen complex at the interface. The changes in the interface properties might be insufficient for their analysis, particularly when the antigen is represented by a small molecule. The secondary antibody labeled with a reporter unit reacts with the affinity complex, then is attached to the antigen analyte. The produced “sandwich” complex composed of antibody–antigen–antibody brings to the surface a reporter unit which produces an amplified output signal. Note that the complex is produced and the reporter unit is bound to the surface only when the antigen analyte is present. Otherwise, the secondary antibody is washed out and the signal from the reporter unit is not produced at the surface. The reporter unit (label) linked to the secondary antibody can be represented by an enzyme (e.g., horseradish peroxidase, HRP), a fluorescent dye, a quantum dot, a Au nanoparticle (NP), or a carbon nanotube (CNT). Some other labels (e.g., magnetic nanoparticles) are not shown in the scheme. The labels can generate an amplified signal in the form of color changes, fluorescence, electric response, microgravimetric response, etc. Respectively, the transducer can be optically transparent, electrically conducted, QCM, etc., depending on the signal generated by the label.

Alternatively, the analyte can be represented by an antigen producing an affinity complex with an antibody immobilized at a transducer surface in the course of immunoassay [9]. Usually, this kind of bioaffinity complex formation requires a secondary reaction with another antibody producing a “sandwich” complex with a reporter label attached to the secondary antibody (Figure 5) [10]. Therefore, the “nonreagent” type of biosensing is not the case in such immunoassays. However, more advanced biosensor operation without the use of secondary antibodies is also possible [11] and will be discussed later. 

DNA sensing can be performed similarly to the immunosensing with the “sandwich” complex produced by complementary DNA molecules at a transducer surface [12]. Primary DNA molecules (usually relatively short oligonucleotides) complementary to a part of the DNA analyte molecules are immobilized at the transducer surface. They produce a double-stranded DNA complex when the analyte DNA is present in a sample solution. Since the double-stranded DNA complex is produced only with a part of the DNA analyte, the rest of the DNA analyte is preserved in the form of a single-stranded DNA available for another complex formation. This part of the DNA analyte is then reacted with another complementary DNA labeled with a reporter unit similar to the secondary antibody used in the immunoassay. The choice of the used reporter unit depends on the signal transducer applied. Overall, the DNA analysis can be conceptually similar to the immunosensing, while technically they might be different depending on the conditions required for the “sandwich” complex formation from antibody–antigen or DNA–complementary DNA species. Notably, the biosensing can be extended to the RNA analysis performed conceptually similar to the DNA sensing. Importantly, some other analytical schemes have been developed for the analysis of DNA/RNA providing higher sensitivity and selectivity, particularly in detecting single-point mismatches in the DNA/RNA molecules [13]. 

Biorecognition units immobilized at a transducer surface are not limited by proteins/enzymes or DNA/RNA molecules and can be represented by more complex biological species, including whole cells or cellular components [14]. In this case, the analyte detection can be based on complex biological processes, and the signal transduction will utilize biological responses to the analyte molecules. The biological responses can be based on gross cellular respiration detected with an O_2_-sensing electrode [15] or with other more sophisticated biological processes. In any case, the biological components (particularly cells) should be immobilized at a transducer surface. 

Nonbiological artificial species, e.g., nanozymes [16], can be used instead of natural biological components. They may have some advantages [17], particularly higher stability, compared to biological species. However, often they suffer from much less specificity and lower catalytic reaction rate than normally can be achieved using biological species. While their biosensing operation might be perfect in model solutions, they may not be specific enough for biosensing in complex real samples having many interferents. Regardless, further research may improve their biosensing operation, keeping their advantages and improving their selectivity and reaction rate. 

## 3. Immobilization of the Biosensing Component at a Transducer Surface

Regardless of the biosensing reactions and signal transduction tools, which can be different, there is a general feature in all biosensors—the biorecognition units must be immobilized at the transducer surface. Methods of chemical or physical immobilization of the biological species can be selected from a very broad variety of options [18], and the choice of the used method frequently depends on the used signal transducer and aimed application. The selected immobilization method should not compromise the biorecognition/biocatalytic features of the biological species. For example, the immobilization technique should not deactivate enzymes by blocking or destructing their active centers. In the case of immunosensing, the immobilized antibody should have an appropriate orientation providing access to their binding sites [19]. Specific alignment of the surface-immobilized biomolecules is rather difficult [20] since proteins/enzymes have multiple identical functional groups involved in the biomolecule attachment and are positioned differently in the protein backbone. While immobilization of biomolecules usually decreases their specificity and activity, this change compared to their soluble state should not be dramatic. 

The immobilization of biomolecules at the signal transducer can be based on different approaches [18]; however, many of them fall into two major categories: (*i*) entrapment in polymer matrices [21], or (*ii*) binding, which can be performed upon covalent bond formation [22] or affinity complex formation [23]. The latter can be in a broad range of binding energies, from 10 to 500 kJ·mol^−1^; thus, it is not surprising that the attachment strength can vary. The immobilization of biomolecules upon their entrapment into polymeric matrices might have a significant problem due to biomolecule leaking, which is dependent on the size of the biomolecules and their charge, as well as on the diameter of pores in the matrices and the polymer charge [24]. Notably, when the entrapped biomolecules and the polymer matrix have the same charges (both positive or both negative), the leakage is facilitated. On the other hand, the opposite charges allow the biomolecules to stay in the matrices with small leakage [24]. It should be noted that all parameters affecting the biomolecule leakage, including the pore size in the matrices, the matrix and the biomolecule charge, can be controlled by the pH of a bulk solution.

## 4. Theoretical Consideration of the Immobilized Biomolecular Systems

### 4.1. Reaction at an Interface

**Figure 6:**
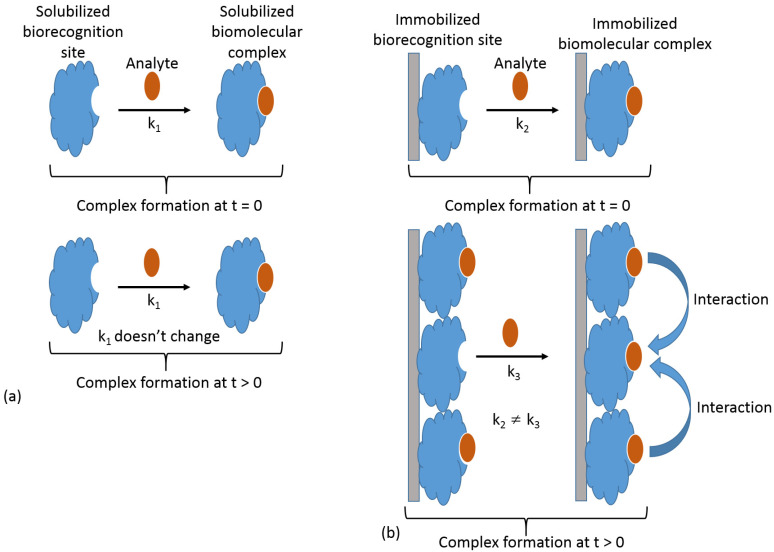
Kinetics of biomolecular recognition processes proceeding in biosensors: (**a**) in a homogeneous solution or (**b**) at a transducer interface. Reaction kinetics remains the same at *t* = 0 and *t* > 0 in the case of a homogeneous reaction; reaction rate constant = *k*_1_ at *t* = 0 and *t* > 0. Reaction rate constant changes according to surface coverage in the case of a heterogeneous process because of the interactions of the neighboring species; reaction constant = *k*_2_ at *t* = 0 and *k*_3_ at *t* > 0.

The biorecognition component immobilized at a signal transduction surface reacts with a soluble analyte; thus, the biosensing process is always a heterogeneous reaction, where one of the reacting components is surface-confined, while the second is delivered by diffusion. In a simplified way, this process can be considered as a bimolecular reaction proceeding at an interface, then being kinetically different from the analogous process under homogeneous conditions when both components are present in a solution. Notably, under homogeneous conditions, the reaction kinetics (i.e., rate constant) remain unchanged regardless of the degree to which the reaction has proceeded, as long as both reacting species are present. In other words, the reaction rate constant is the same at the beginning of the reaction process, *t* = 0, and during the reaction time, *t* > 0 (Figure 6a). On the other hand, in the case of the heterogeneous reaction, the individual biomolecule sites immobilized at the surface are not independent, and the reaction (particularly complex formation) of one site affects the reactivity of neighboring sites (Figure 6b), thus changing the reaction kinetics in the course of the analyte reaction with the immobilized biorecognition sites. This phenomenon originates from the high local concentration of the reacting species at the modified surface. This local concentration is dramatically higher than a possible concentration of the same species in a solution.

**Figure 7:**
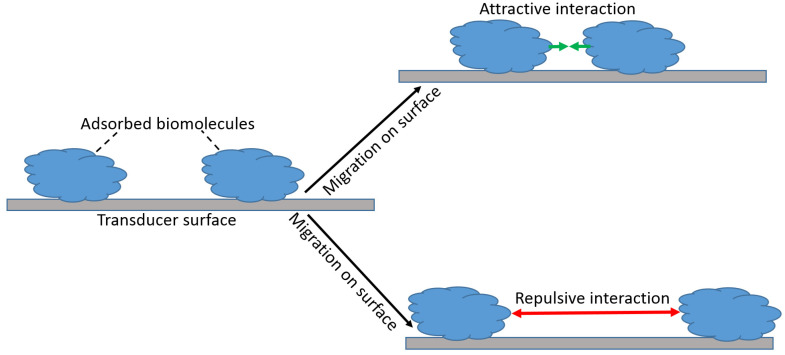
Migration of biomolecule adsorbate with attractive or repulsive interactions.

The attractive and repulsive forces between the complex of the biorecognition units and analyte species can extend up to 50 nm, and their intensity may vary significantly. For simplicity, we can consider the biorecognition units as adsorbed and surface-mobile species. Then, the biorecognition units adsorbed at the signal transducer surface may be localized at their initial positions for the entire time of the biosensor operation. This is particularly correct when they are attached (including covalent binding) to the surface. Alternatively, they can migrate along the surface, which is the case of their weak adsorption (Figure 7). Obviously, this migration is possible only when the activation energy for a weakly bound biomolecule species is less than the binding energy to the support, and it is not the case when the biomolecules are immobilized by the formation of covalent bonds or strong chemisorption (e.g., chemisorption of thiol groups on a Au surface). 

The signal transducer surface may have some roughness (sometimes very significant). If the immobilized biomolecules (particularly large proteins, especially antibodies) have a size comparable to the heterogeneity of the nanorough surface, the adsorbed biospecies will interact with different planes of the surface. Since each plane of a given orientation has a different activity for the biospecies adsorption, it is expected that the biomolecules may move from the primary adsorption place to the plane providing stronger adsorption energy and more stable immobilization. In the case that the surface roughness is not of the size of the adsorbed biomolecules (which means it is of microsize, much bigger than the nanosize of the adsorbed biomolecules), the adsorption energy is elevated with increased coverage due to dipole–dipole interactions between the adsorbed biomolecules and new incoming molecules. For an ideal, nonmobile, simple adsorbed layer, the decrease in the heat of adsorption is linear with the biomolecule coverage. In a mobile layer the heat of adsorption remains nearly constant initially and then shows a step inflection at ca. 50% coverage [25]. This indicates that the result of high mobility is to minimize lateral biomolecule interactions. The effects briefly explained here are quantitatively considered as different types of adsorption isotherms [26]. 

### 4.2. Range of Forces Affecting Adsorbed Biospecies

**Figure 8:**
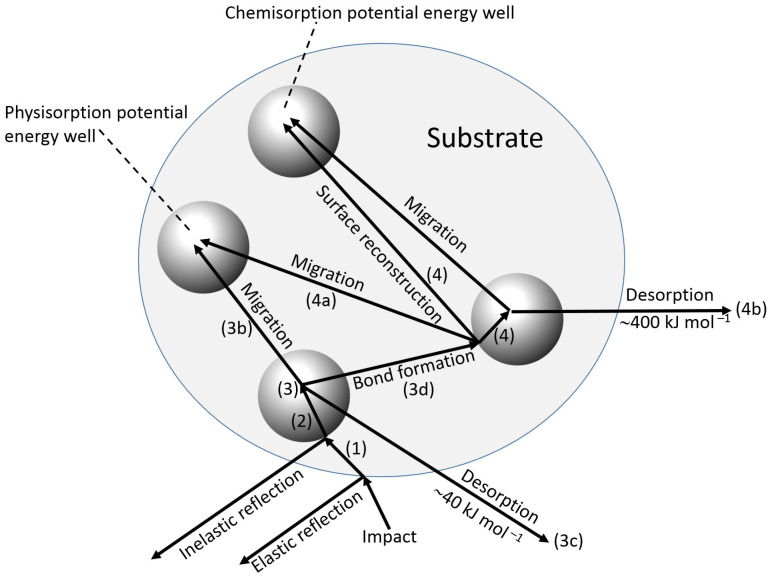
The sequence of events at a surface leading to chemisorption.

During the adsorption of a biorecognition molecule on a signal transducer surface, the primary collision of the biomolecule with the surface can be followed by a number of different processes shown schematically in Figure 8 and listed below [25]:

1. Reflection back into the bulk solution of the adsorbate, with (inelastic) or without (elastic) transfer of energy.
2. Transfer of energy (inelastic collision) such that the molecule is unable to “climb” out of the potential well at the surface and is in an excited physisorbed state, which is associated with comparatively weak forces (e.g., van der Waals), and a low enthalpy of adsorption of ca. 40 kJ·mol^−1^.
3. Subsequent possible processes:
  (a) Further loss of energy to the surface at the same site.
  (b) Migration over the surface with loss of energy at other sites.
  (c) Desorption with a gain in energy from the adsorbent.
  (d) Transfer to the chemisorbed state, either at the initial site or after migration.

Chemical adsorption is associated with the formation of a chemical bond with a typical enthalpy change of ca. 400 kJ·mol^−1^.

4. Once chemisorption has occurred, further possibilities exist.
  (a) Migration of the chemisorbed species.
  (b) Desorption from the chemisorbed state.
  (c) Further chemisorption giving multiple attachments.
5. Surface reaction may take place between the incoming molecule and another species already adsorbed, but not directly involve the substrate.

Chemisorption [27] has a large enthalpy of adsorption, greater specificity of orientation and more stable layers than physisorption [28]. Nonetheless, on a surface containing many sites capable of physical and chemical adsorption, a mixed population of adsorbed species is still likely to accumulate, whose nature varies with temperature and rate of deposition. Once the biorecognition surface has been created, its reaction with the analyte will not only be homogeneous, but will also involve chemisorption.

## 5. Methods of Immobilization

The immobilization of the biorecognition element can be performed in one of two ways. The biorecognition molecules can be attached directly to the signal transducer surface, or else a “carrier substrate” (e.g., a polymer film) can be modified with the biorecognition molecules, which is subsequently, or sometimes simultaneously, deposited on the transducer surface.

### 5.1. Physical Adsorption

The simplest deposition of biorecognition molecules onto a signal transducer surface is their physical adsorption. The most popular adsorbent for the physical immobilization of enzymes has been based on carbon electrodes [29] (e.g., glassy carbon electrode [30], pyrolytic electrode [31], buckypaper electrode [32], etc.), then used for electrochemical biosensing. Polystyrene has been frequently used for the deposition of biomolecules (particularly antibodies), then utilized for optical analysis in various biosensors [33]. While the advantage of this approach is its simplicity, it still suffers from weak stability resulting in fast leaching of the biomolecules from the biosensor surface. A simple way to protect the biomolecule layer from leaching has been the use of an analyte-permeable membrane covering and protecting the biomolecule film loaded on the transducer surface [34]. 

Although physical adsorption is rather simple, it can also be the least efficient for biosensing since small control of the orientation or site of attachment is possible without modification of the surface itself or changing the activity of the adsorbed biomolecule species. This has been illustrated by comparing human immunoglobulin G (hIgG) adsorbed on SiO_2_ and on an aminosilane-treated SiO_2_ surface [25]. Both surfaces demonstrated a comparable amount of the adsorbed hIgG antigen; however, the amount of the bound antibody, anti-hIgG, per antigen molecule was significantly different. While the hIgG antigen adsorbed on the untreated SiO_2_ surface reacted with about three anti-hIgG species, another hIgG antigen adsorbed on the amino-silanized SiO_2_ surface reacted with about six molecules of anti-hIgG species. This can be explained [25] by the different orientations of the hIgG antigen molecules at the support surface. In other words, a similar amount of biorecognition molecules adsorbed at a signal transducer surface may result in different bioaffinity in the case of immunosensing or biocatalytic activity in the case of enzyme-based catalytic processes. In general, the adsorption of immune proteins (antigens or antibodies) on a transducer interface is not always appropriate for immunosensing. If the transducer surface is not fully blocked with the adsorbed immune proteins, the vacant areas of the sensing surface may adsorb other biospecies present in a sample, then compromising the biosensing selectivity. This problem is particularly serious if the biosensing is based on labelless measurements. In this case, the immunosensing response originates from the loading of all possible species on the transducer surface: increase in mass in the case of QCM measurements, refraction index at the surface in the case of SPR measurements, electron-transfer resistance in the case of Faradaic impedance measurements, charge associate with the surface in the case of ion-selective field-effect transistor (ISFET) measurements, etc. Since all these measurements rely on the total changes in the interface parameters, the result might be compromised by nonspecific adsorption, which always results in the loss of selectivity. While the problem caused the nonspecific adsorption exists for all kinds of immobilization, it is particularly severe in the case of the physical adsorption of the immune proteins. A densely packed surface immobilization of immune proteins may not suffer from nonspecific adsorption, but nevertheless, it is often still not effective for antibody–antigen complex formation because of steric hindrance of the binding areas in the protein structure. Conversely, a widely spaced packing usually allows nonspecific interactions with the underlying surface to occur, and false-positive signals may be recorded.

### 5.2. Physical Retention in Polymer Matrices

**Figure 9:**
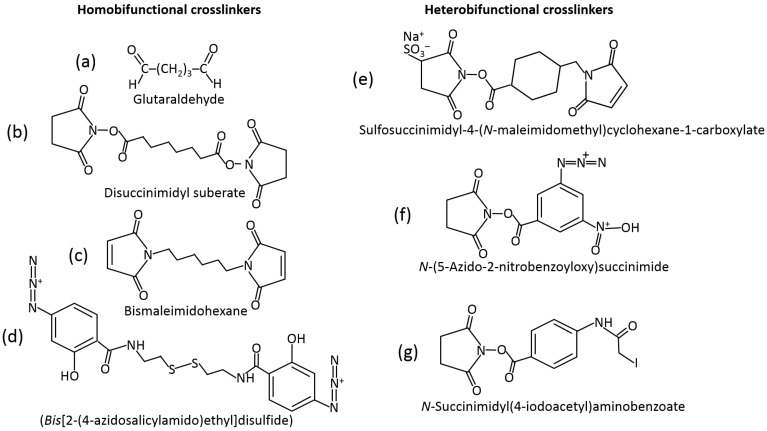
Bifunctional reagents: (**a**–**d**) homobifunctional—containing two identical reacting groups for binding to the same functional groups of biomolecules; (**e**–**g**) heterobifunctional—containing two different reacting groups for binding to different functional groups of biomolecules. The following reacting groups are shown in the examples given: (**a**) aldehyde groups reacting with amino groups, (**b**,**e**,**f**,**g**) active ester groups reacting with amino or hydroxyl groups, (**c**,**e**) maleimide and (**g**) iodoacetyl groups reacting with thiol groups, and (**d**,**f**) azido groups used for click-chemistry reactions. The spacers separating the reacting groups can be flexible (**a**–**d**) or rigid (**e**–**g**), thus resulting in different attachment modes of biomolecules. The spacer length can be different, short or long, and some spacers can be cleavable due to S–S bonds (**d**) or other chemically or photochemically cleavable units.

The most frequently used method of immobilization of biomolecules for various applications, including biosensing, is their entrapment into polymer matrices producing a hydrogel upon the polymer cross-linking [35,36,37]. The polymers used (e.g., starch [38], hyaluronic acid [39], chitosan [40], alginate [41], cellulose [42], gelatin [43], collagen [44], etc.) and cross-linking reagents [45] are really numerous. They might be exemplified with amino-functionalized polymers cross-linked with glutaric dialdehyde resulting in the Schiff-base bonds at the position of amino groups [46]. Many other cross-linking reagents (homo- [47] and hetero-bifunctional [48]) have been used, and their examples are shown in Figure 9. The majority of the cross-linking reagents target amino- (–NH_2_), carboxyl- (–COOH), hydroxyl- (–OH) or thiol groups (–SH) in polymers. Carboxyl-functionalized polymers (e.g., alginate) can be cross-linked with divalent or trivalent metal cations (e.g., Ca^2+^ or Fe^3+^) yielding hydrogels [49,50]. The Ca^2+^-cross-linked alginate hydrogel with interpenetrating polyvinyl alcohol can be additionally cross-linked, decreasing its porosity with diboronic acid derivatives [51]. 

**Figure 10:**
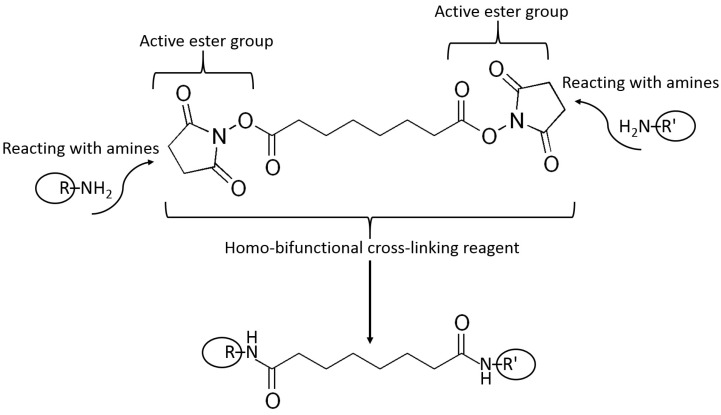
A homobifunctional cross-linker containing two active ester functions used to bind biomolecules by reacting with their amino groups and producing amido bonds. Note the long and flexible spacer between the reacting groups. Many other similar cross-linkers are available with various structures and length of the spacer connecting the reacting groups.

**Figure 11:**
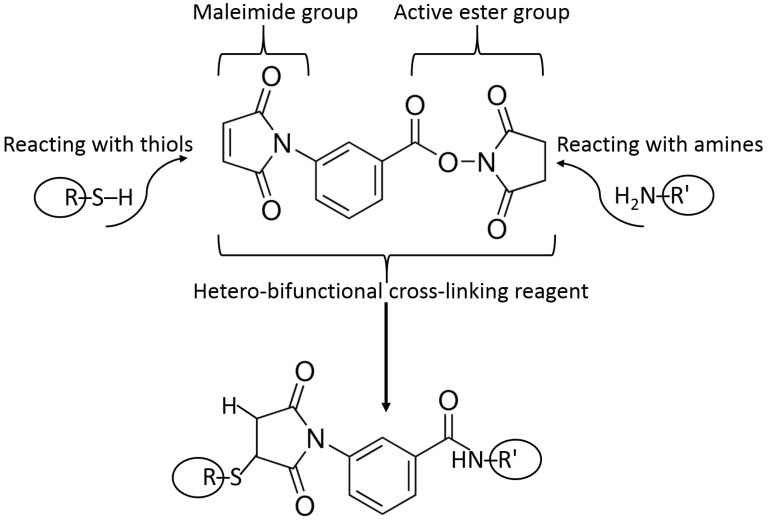
A heterobifunctional cross-linker containing a maleimide function for binding to thiol groups of biomolecules and an active ester function for binding to amino groups of biomolecules. Note the short and rigid spacer between the reacting groups. Many other similar cross-linkers are available with various structures and lengths of the spacer connecting the reacting groups.

Example reactions for biomolecule binding using homobifunctional and heterobifunctional cross-linkers are shown in Figure 10 and Figure 11, respectively. Note that homobifunctional reagents have the same functional groups at both sides of the spacer, while heterobifunctional cross-linkers possess different functional groups at both sides.

Many different polymers have been used as matrices for immobilization of biomolecules, then used for biosensing, including gelatin, agar, cyclodextrins, polypyrrole, polyaniline, polyvinylchloride, polyvinylalcohol, polyvinylpyridene, polyacrylamide, chitosan, alginate, etc. Some of these polymers are synthetic (e.g., polypyrrole, polyaniline, etc.), and some others are natural (biological) (e.g., chitosan, alginate, etc.). The choice of the polymer matrix depends on the kind of biomolecules entrapped and the application targeted. In many biomedical applications, the biocompatibility of the polymer is important [52]. The stability and preserved activity (biocatalytic or biorecognition) of the entrapped biomolecules are highly important. They should not lose the biological activity upon immobilization and should not leak from the polymer matrices, providing stability to the biosensors. On the other hand, the polymer matrices should allow penetration of the analyte species to the biosensing components. For aqueous or water-containing applications the hydration properties are also important. A newer approach has been to use film-forming emulsion polymers [53], where pH, isotonicity and hydration properties can be tailored to the biorecognition system and application. This means that a similar immobilization protocol can be employed, irrespective of whether it is an enzyme, antibody or whole cell. The biosystem-containing emulsion can be prepared in aqueous media even though the film becomes insoluble after formation.

Another powerful approach is based on the electrochemically stimulated formation of hydrogels deposited on an electrode surface with physically entrapped biomolecules. This is particularly convenient for cross-linking of alginate with Fe^3+^ cations produced upon electrochemical oxidation of Fe^2+^ cations [54,55]. Note that the Fe^2+^ cations present in a solution are not active for the alginate cross-linking, while the Fe^3+^ cations produced at the electrode surface are effective cross-linkers for the alginate polymers. Then, the Fe^3+^-cross-linked alginate hydrogel produces a polymer matrix entrapping biomolecules from the solution. Later, the Fe^3+^ cations can be exchanged by Ca^2+^ cations, if needed [24].

Many other approaches to producing matrices with biomolecules entrapped have been suggested and applied. They include the formation of sol–gel silicate glasses [56], carbon paste [57], etc. While the sol–gel glasses might be convenient for optical biosensing, carbon paste electrodes are applicable to the electrochemical biosensing.

### 5.3. Surface Modification

Most of the materials composing signal transducers do not have chemical functional groups for producing direct covalent coupling with biomolecules for their immobilization. Therefore, in a standard approach, the transducer surface first reacted with organic species, which yielded functional groups for further covalent binding of the biomolecules. The majority of the organic species are polymers physically adsorbed at the transducer surface. The most common example is polyethylenimine, which is strongly adsorbed on many solid materials and offers amino groups for further covalent binding of the biomolecules [58]. Other examples of the adsorbed polymers include maleic anhydride copolymers [59], methacrylic acid/anhydride copolymers [60] and their derivatives. In general, the adsorbed polymers introduce to the transducer surfaces’ free –NH_2_, –SH, –COOH, etc., groups. Such supports are deposited over the transducer surface as a thin layer. This layer usually does not compromise the operation of the signal transducer, e.g., the adsorbed polyethylenimine does not introduce meaningful electron-transfer resistance at electrodes used in electrochemical signal transduction. The pendant reactive groups, through which the biorecognition molecule will be attached, can be added to the polymer prior to its adsorption by derivatization of the monomer and copolymerization, or by direct reaction with the preformed polymer.

**Figure 12:**
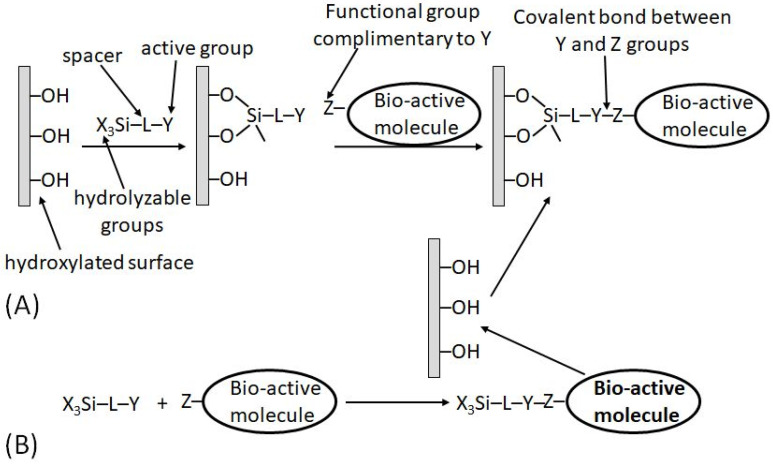
(**A**) Immobilization of a bioactive molecule onto a silane-derivatized substrate. (**B**) Immobilization of a silane-derivatized bioactive molecule.

In a different approach, the functional groups pre-existing in the transducer material (e.g., hydroxyl groups, –OH, available in glass, glassy carbon or indium tin oxide (ITO), electrode) can be reacted with silane coupling agents of the type X_3_SiLY [18], where X is a hydrolyzable group, L is a spacer and Y is an active functional group (or one capable of being transformed into a functional group). The thermal and hydrolytic stability of the resulting linkage is high on such supports. Two approaches are possible in designing the desired surface modification:

(a) The coupling agent is attached first to the support and then reacted with the macromolecule requiring immobilization (Figure 12A);
(b) The macromolecule-silane complex is formed first and then reacted with the transducer surface (Figure 12B).

It should be noted that the second approach (b) is not applicable to biomolecules and can be used for the immobilization of synthetic bioactive molecules only, which can tolerate the reaction in nonaqueous solvents. This limitation is quite obvious because the hydrolyzable functions (X) in the silane reagents are rapidly destroyed in aqueous solutions; thus, all reactions with participation of the silane reagents are always performed in nonaqueous solutions (usually in dry toluene). When the schematically shown “bioactive” molecule is indeed represented with a real biomolecule (e.g., protein/enzyme), the immobilization pathway (a) is the only possibility.

While both synthetic/immobilization approaches may result in the same result schematically shown, in reality, the reaction output might be significantly different because of a large difference in the reaction conditions leading to possible side processes. Depending on the exact reaction conditions, the coverage might be close to a monolayer structure or can be a polymeric film.

**Figure 13:**
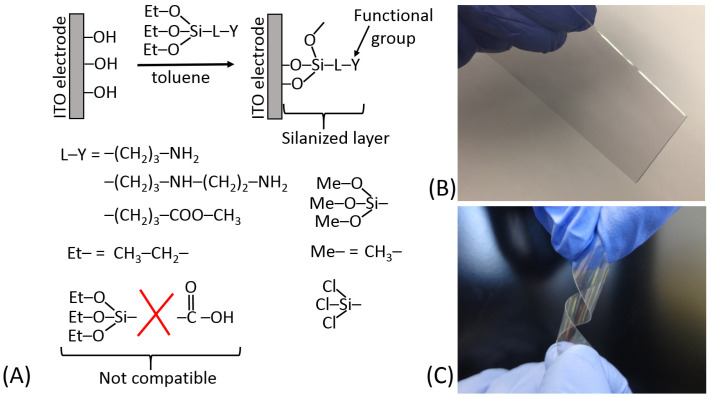
(**A**) Schematically shown silanization of an indium tin oxide (ITO) electrode used in electrochemical biosensors. Note that glass surfaces can be modified similarly, while being used in optical biosensors. (**B**) The ITO electrode deposited onto a glass slide as a thin conducting film (note a rigid support for the ITO electrode). (**C**) The ITO electrode deposited onto a polymer film as a thin conducting film (note a flexible support for the ITO electrode).

**Figure 14:**
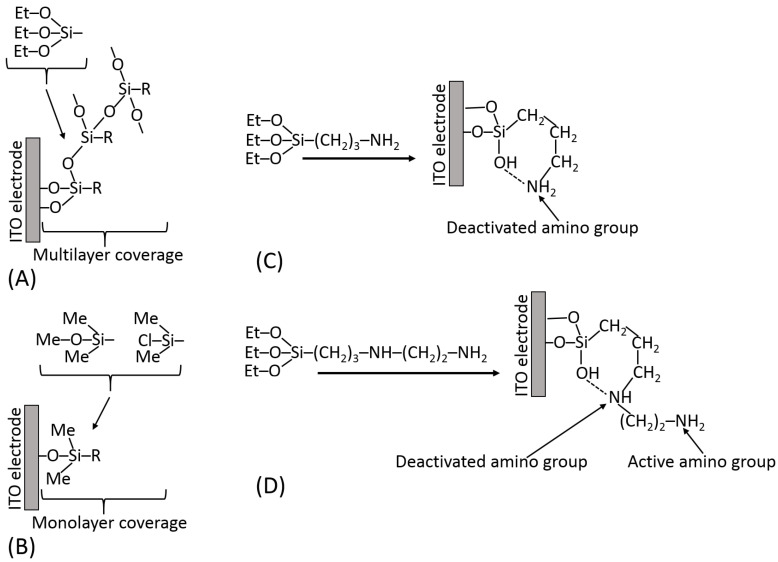
(**A**) Multilayer coverage of an ITO electrode upon silanization with a silane with three hydrolyzable groups performed in protonic solvents (e.g., ethanol) or in aprotic solvents (e.g., toluene) with traces of water. (**B**) Strictly monolayer coverage of an ITO electrode using silane with one hydrolyzable group. (**C**) Deactivation of the terminal amino group upon formation of a cyclic structure in the silane layer. (**D**) Preserving an active terminal amino group in the silane layer with two amino groups in the silane molecule.

Figure 13A schematically shows the silanization of an indium tin oxide (ITO) electrode and exemplifies different hydrolyzable groups (Me–O–; Et–O–; Cl–). It should be noted that some functional groups (particularly carboxylic) are not compatible with the hydrolyzable groups; thus, they should be generated on the silanized surface after the silanization reaction from the appropriate precursor functions, e.g., from the ester groups. The surface modified by the silanization process can be represented by different materials; for example, ITO electrodes can be deposited as thin films on glass or flexible plastic, as shown in Figure 13B,C, respectively. The silane reagents containing three hydrolyzable groups have a tendency to produce polymeric films unless strictly dry solvents (e.g., very dry toluene) are used for the silanization process [18] (Figure 14A). The monolayer coverage can be produced with a silane reagent containing only one hydrolyzable group (Figure 14B). However, the only hydrolyzable group could be easily deactivated prior to the silanization process, thus resulting in poor surface modification. The active functional groups located at the end of the spacer (specifically –NH_2_ groups) can be deactivated due to their reaction with other groups at the surface (Figure 14C). In order to keep them free and active, some structural features should be provided using linkers with additional groups preventing their deactivation (Figure 14D). Silanization of hydroxylated surfaces is not the only available method for their modification—many other linkers can be used, reacting at different conditions and resulting in different surface structures.

**Figure 15:**
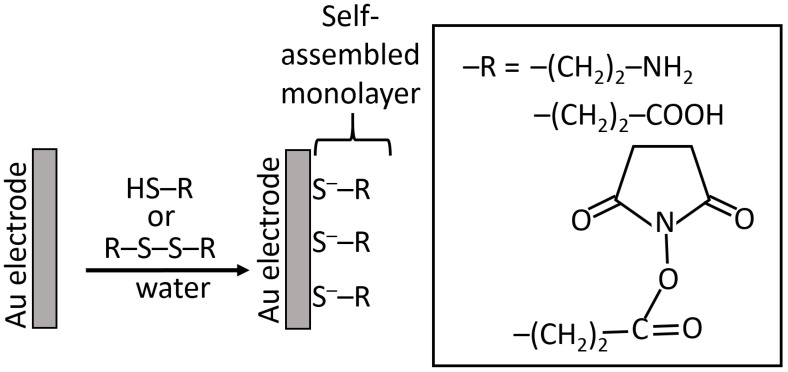
Self-assembly of thiol/disulfide molecules on a Au electrode, then introducing various functional groups (amino-, carboxyl- or active ester for covalent immobilization of (bio)molecules).

While silanization of Au and Pt electrodes is possible using an electrochemically produced metal oxide layer for the reaction with silane reagents [61], another method based on the self-assembly of sulfur-containing molecules is much more convenient and frequently used for the electrode modification [18,62]. Chemisorption of the sulfur-containing molecules (usually thiols [63] or disulfides [64], rarely sulfides [65]) results in the formation of well-organized monolayers with terminal functional groups (frequently amino or carboxyl groups, but sometimes others) available for covalent immobilization of various (bio)molecules, including proteins/enzymes, DNA, RNA, etc. (Figure 15) [18]. The use of cysteamine (thiol molecules) and cystamine (disulfide molecules) became a commonly used method for Au (and some other noble metals) modification. Since this method became a piece of general knowledge, the original reference introducing this modification technique is usually omitted [66]. Notably, the monolayers produced upon chemisorption/self-assembly of thiols and disulfides have similar structures obtained by the dissociation of thiol groups and splitting disulfide groups [64], then yielding the same adsorbed species. The kinetics of their adsorption is different, requiring a longer time for the disulfides compared to thiols [66].

Many other compounds of the structure Y(CH_2_)*_n_*X having two terminal functional groups separated by a hydrophobic chain (spacer) will organize themselves at a surface according to the reactivity of the functional groups [18]. If Y is chosen to react with the surface, then an ordered layer, including the spacer, can be formed in one step. Different functional groups can be introduced provided that they will not compete with the Y head group for coordination with the substrate, and they will not be so large that they prevent close packing of the hydrocarbon chains. An example of such immobilization might be with an aromatic anchor group (e.g., pyrene) chemisorbed on a buckypaper electrode due to π–π interaction with carbon nanotubes in the buckypaper [67].

It should be mentioned that some biomolecules may have thiol groups in their structure (e.g., thiol-derivatized oligonucleotides or cysteine-contained oligo/polypeptides) [68]; thus, they can be directly chemisorbed on Au electrodes without a need of preliminary deposition of sulfur-containing bifunctional reagents.

### 5.4. Organizing Biosensor Interfaces

Even having achieved the successful immobilization of the biorecognition molecules, the task of applying the layer to the sensor surface may not be complete. In the examples given above, either the immobilization reagents or the polymer matrix has to be deposited accurately and reproducibly onto the surface of the electrode. The thickness and diffusion characteristics of this layer will determine the character of the final response signal.

In the field of electronics, deposition and definition are performed to precise limits of accuracy. Thick and thin film technologies have become tools in the fabrication of miniaturized electronic circuits. Several different techniques are available [25], all of them suitable for adaptation, for the deposition of layers containing the biorecognition element onto a transducer.

Screen printing [69] is a thick film method that can be used to deposit a range of electrode materials (including carbon, Ag/AgCl reference electrode and noble metals) as well as the biorecognition layer, mediators, or protective overlayers. Typically, the accuracy of the deposition is controlled to within 500 μm, although with certain materials, 100 μm has been reported.

Ink-jet printing [70] is able to dispense single drops or up to 50 000 drops per second, of a conducting solution, such as a buffered protein solution. The droplet size can be selected between 0.3 and 1.5 nL, resulting in 1–5 μm layers depending on the surface tension of the liquid and the hydrophobicity and absorbency of the substrate on which it is printed.

Conventional photoresists [71] can be patterned with a resolution approaching 250 nm using a 350–450 nm light source, or less than 100 nm using an electron beam technique. With protein/polymer layers, however, the practical limitations of photolithography are estimated to be greater than 150 μm because of the light scattering induced by the protein.

## 6. Signal Transduction in Biosensors

### 6.1. Amperometric Signal Transduction—Theoretical Consideration and Practical Applications

Amperometric signal transduction is the most frequently used method in enzyme-based biosensors [72]. The steady-state current (*I*_d_) measured at a chemically modified (usually with bound enzyme molecules and electron-transfer mediators) electrode under diffusion control can be expressed by Equation (1) [25]:

(1) $$I_{d}=\frac{nFD\;\left[S\right]}{d}$$

where *n* is the number of electrons per the enzyme-substrate oxidation (or reduction), *d* is the diffusion layer thickness, *D* is the diffusion coefficient of the measured species (enzyme substrate) in the layer, and [*S*] is the substrate (analyte) concentration.

**Figure 16:**
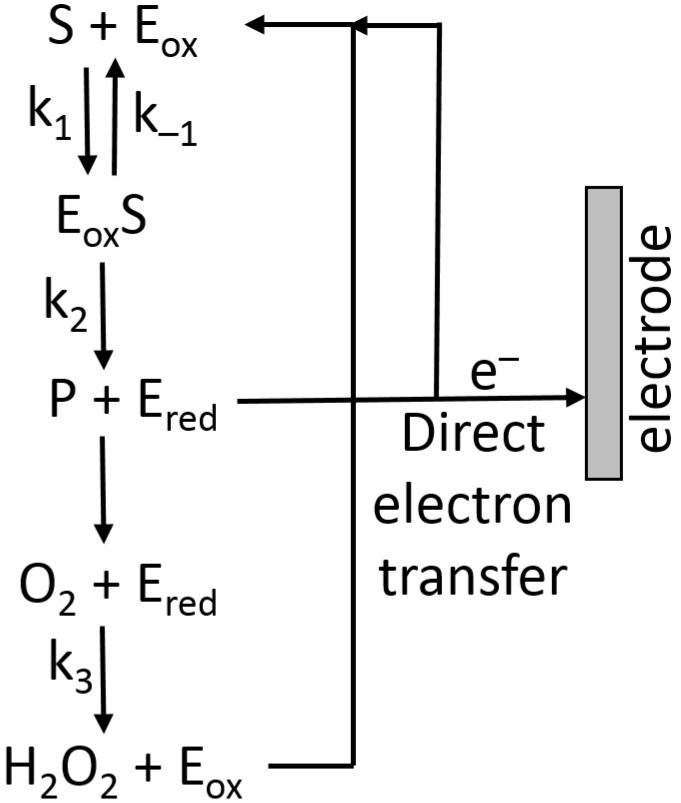
Kinetic steps of oxidase enzyme catalysis.

The current measured at an electrode under potentiostatic control can be related to the kinetics of the enzyme reaction and depends on the substrate concentration, if the concentration is not too high to result in the enzyme saturation (being in the dynamic concentration range). The rate of an enzyme-catalyzed reaction is given by Equation (2) [25]:

(2) $$\frac{d\left[S\right]}{dt}=\frac{k_{2}\;\left[E_{0}\right]\;\left[S\right]}{K_{M}+\left[S\right]}$$

where *K*_M_ is the Michaelis–Menten constant, and *E*_0_ the total enzyme concentration (in the case of the immobilized enzyme, it is the total amount of the enzyme bound to the modified electrode), *k*_2_ is the rate constant defined in Figure 16.

Assuming that the reoxidation of the enzyme (Figure 16) is the fast step and it does not limit the reaction, then, at the simplest level, the current at an electrode under enzyme kinetic control could approximate to Equation (3) [25]:

(3) $$I_{k}=\frac{nFdk_{2}\;\left[E_{0}\right]\;\left[S\right]}{K_{M}+\left[S\right]}$$

This gives a maximum current response *I*_max_ when [*S*] ≫ *K*_M_, expressed in Equation (4):

(4) $$I_{\max}=nFdk_{2}\;\left[E_{0}\right]$$

where *n* is the number of electrons per the enzyme-substrate oxidation (or reduction), *F* is the Faraday number, *d* is the diffusion layer thickness, *E*_0_ is the total enzyme concentration, and *k*_2_ is the rate constant defined in Figure 16 [25].

Finally, the diffusion-limited current (*I*_d_) could be described by Equation (5):

(5) $$I_{d}=\frac{I_{\max}\;D\;\left[S\right]}{d^{2}k_{2}\;\left[E_{0}\right]}$$

and the current limited by the enzyme kinetics (*I*_k_) expressed by Equation (6) [25]:

(6) $$I_{k}=\frac{I_{\max}\;\left[S\right]}{K_{M}+\left[S\right]}$$

which suggests that, for a given enzyme concentration, *I*_d_ decreases with increasing *d* and increases with increasing *D*, whereas *I*_k_ increases with increasing *d* and is independent of *D*. This presumes that the concentration of all species throughout the layer is the same and that consumption of any substrate at the outer surface does not lead to a depletion near the electrode (where the measured reaction is taking place). In practice, a nonlinear enzyme reaction is more probable and an accurate calibration of the electrode must be taken into consideration. Several models have been devised that can predict the concentration gradients through the immobilized layer [73,74]. The need to consider concentration gradients and cosubstrate limitations is important in ensuring that the biosensor performs in the way in which it was intended.

### 6.2. Electron-Transfer Mediators

**Figure 17:**
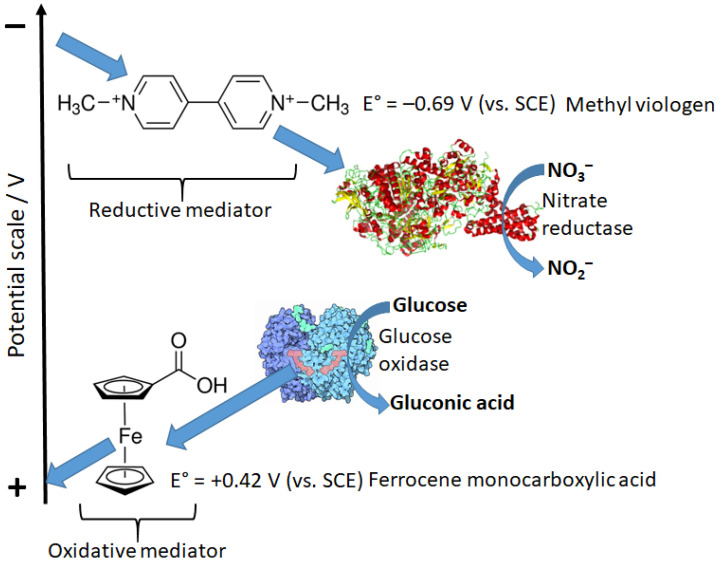
Examples of the oxidative and reductive soluble mediators shuttling electrons between electrodes and oxidative or reductive enzymes. Mediators can be used as “charge transfer messengers” between electrodes and enzymes.

The reaction pathway shown in Figure 16 includes the direct electron-transfer step [75] from the reduced enzyme, E_red_, to an electrode, recycling the enzyme back to its oxidized form, E_ox_. This would be an excellent way to couple a biocatalytic process with an electronic signal transducer. Unfortunately, the majority of enzymes, particularly oxidases (e.g., glucose oxidase), are not capable of this direct electron transfer because it should proceed over a long distance, which does not allow fast electron transfer [76,77]. This can be explained according to the Marcus electron-transfer theory [78,79], which predicts an exponential decrease in the electron-transfer rate with the increasing distance. Note that the long distance for this electron transfer originates from the location of enzyme active centers deeply in the protein body. In order to solve the problem, electron-transfer mediators (sometimes named relays) shuttling electrons between enzyme active centers and an electrode surface should be used [18,80]. Depending on the direction of the electron transfer: from an oxidative enzyme to an electrode surface (e.g., glucose oxidase [81]) or from an electrode to a reductive enzyme (e.g., glutathione reductase [82] or nitrate reductase [83]) mediators with different redox potentials can be used (Figure 17).

**Figure 18:**
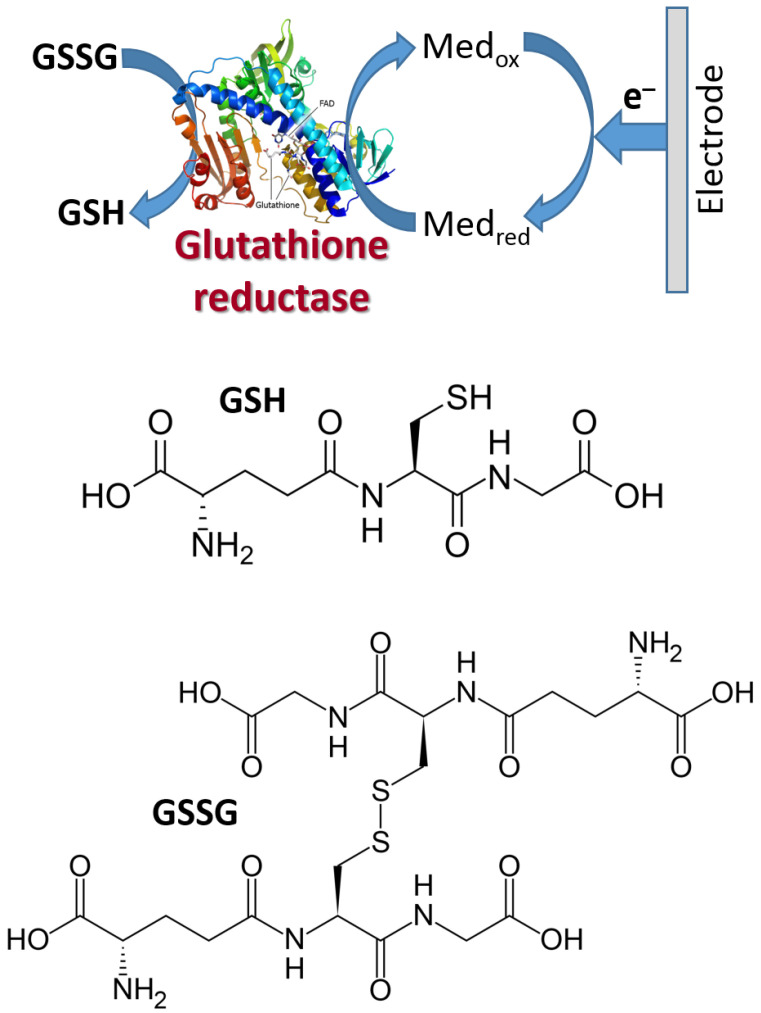
Example of mediated reductive bioelectrocatalysis.

Figure 18 shows schematically the reductive biocatalytic process mediated with a redox mediator with a very low (negative) potential capable of electron transfer from an electrode to glutathione reductase enzyme resulting in bioelectrocatalytic reduction of oxidized glutathione (GSSG) yielding its reduced form (GSH). The cathodic (reductive) current can be used to analyze the GSSG concentration. Note that the electrochemical process of this kind, proceeding at low potentials, should be performed under strictly anaerobic conditions (in the absence of oxygen) because oxygen will compete with the mediator at the negatively polarized electrode. The mediator can be exemplified with methyl viologen (Figure 17) or the immobilized derivative of viologen.

**Figure 19:**
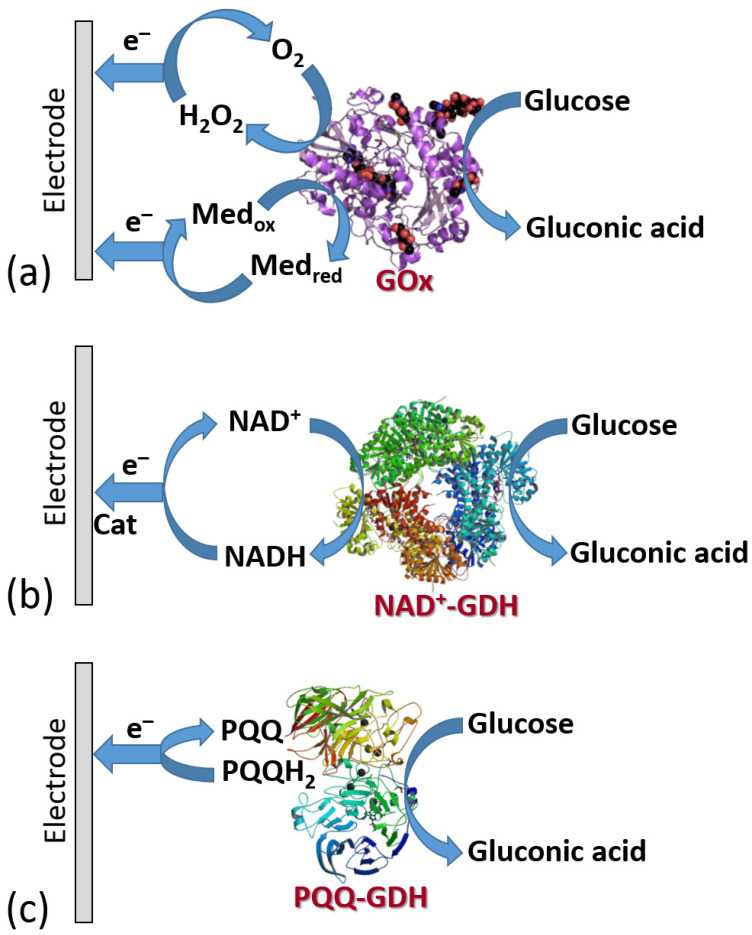
Bioelectrocatalytic processes involving different redox enzymes: (**a**) flavin adenine dinucleotide (FAD)-oxidases (exemplified with GOx), (**b**) NAD^+^/NADH-dehydrogenases (exemplified with NAD^+^-glucose dehydrogenase, NAD^+^-GDH); note that a catalyst, Cat, is usually required for recycling the NAD^+^/NADH cofactor), and (**c**) PQQ-glucose dehydrogenase (PQQ-GDH). All three enzyme classes are exemplified here with the enzymes oxidizing glucose.

**Figure 20:**
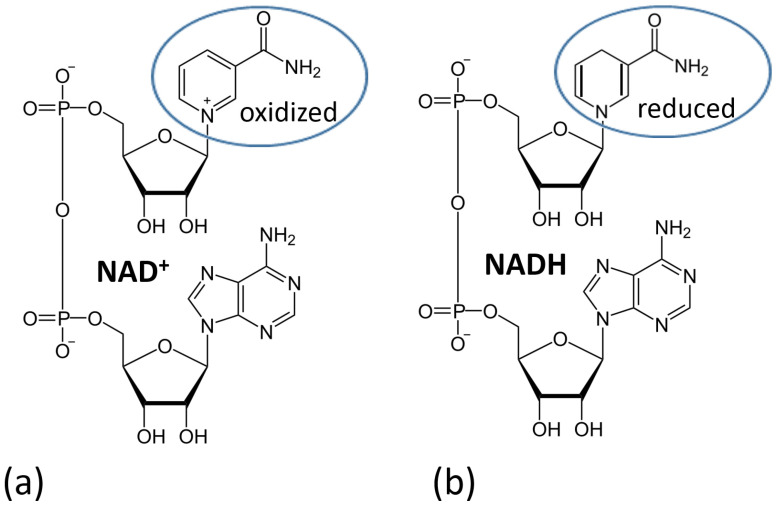
NAD^+^/NADH cofactor structure in the oxidized (**a**) and reduced (**b**) states. Note that this cofactor is used by NAD^+^/NADH-dependent dehydrogenases.

Figure 19 shows the three classes of enzymes most frequently used in amperometric enzyme electrodes. Oxidases (exemplified with glucose oxidase (GOx)) usually operate with redox mediators (Med) transporting electrons from the enzyme active centers to electrodes. The mediator is cycled between the reduced and oxidized states (Med_red_ and Med_ox_), then exchanging electrons with the enzyme and electrode. Also, these enzymes can reduce O_2_ yielding H_2_O_2_ which can be also detected electrochemically at an electrode surface. Note that the O_2_ biocatalytic reduction usually dominates, being more favorable kinetically; thus, the mediated electron transfer is usually realized under anaerobic conditions. NAD^+^-dependent enzymes (exemplified with NAD^+^-glucose dehydrogenase, NAD^+^-GDH) require NAD^+^/NADH cofactor to recycle electrons (see the cofactor structures in both oxidation states in Figure 20). However, the reduced form of this cofactor (NADH) is very difficult for reoxidation at most of the electrode materials and needs a special catalyst (usually quinonoid species immobilized at the electrode surface) [84,85]. Another example, pyrroloquinoline quinone-dependent glucose dehydrogenase (PQQ-GDH) can operate without any mediator with direct electron transfer to an electrode surface [86]; however, this process requires careful selection of the electrode material (e.g., made of buckypaper composed of compressed carbon nanotubes) and appropriate immobilization of the enzyme molecules at the electrode surface. Notably, NAD^+^/NADH- and PQQ-dependent enzymes are not O_2_-sensitive; thus, they can transport electrons in the mediated or direct electron transfer to electrodes in the presence of O_2_ and without the production of H_2_O_2_.

**Figure 21:**
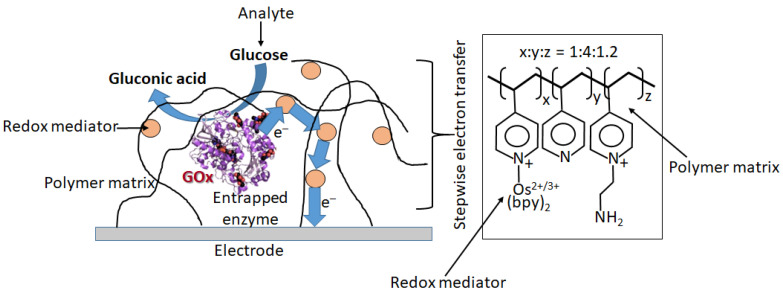
Bioelectrocatalytic glucose oxidation by the system immobilized at an electrode surface. The system includes a polymeric matrix bound to the electrode surface and composed of a cross-linked polymer network with pendant redox mediator groups. The GOx enzyme is physically entrapped into the polymer matrix. The redox mediator used in the system is [Os(2,2′-bipyridine)_2_]^+^ covalently bound to the (poly)4-vinylpyridine network.

When the bioelectrocatalytic process requires the participation of electron-transfer mediators (this is the case for a majority of redox enzymes, particularly for GOx), the mediator can operate as a diffusional or immobilized species [18]. However, practical applications obviously require the immobilized mediator to exclude its presence in the analyzed solution, especially in biomedical applications. There are numerous approaches to the immobilization of redox mediators, usually being coimmobilized with redox enzymes at electrode surfaces [18]. Figure 21 shows schematically one of the options, where glucose oxidase (GOx) is physically entrapped into a polymer matrix functionalized with many pendant redox mediator groups [87]. The electron transfer is realized through multistep electron hopping from one mediator group to another until it reaches the electrode surface.

A mediator completes the enzyme redox cycle and returns to its initial form. In this situation, it is always recycled, and should not cause the reaction to become limited by its depletion. Unfortunately, this is not always the case, and cosubstrate (mediator) limitations may exist.

### 6.3. Electrodes Functionalized with Oxidase Enzymes and Some Other Redox Enzymes

The very first pioneering report on an enzyme-based biosensor was published in 1962 by Clark and Lyons from the Children’s Hospital of Cincinnati [88]. The glucose oxidase (GOx) enzyme was immobilized on a membrane located near a Pt electrode polarized at +0.6 V (vs. saturated calomel electrode, SCE). The biocatalytically produced H_2_O_2_ in the presence of O_2_ (but in the absence of a mediator) was oxidized electrochemically, and the measured anodic current was proportional to the glucose concentration (Figure 19a). It was the first described first-generation biosensor based on direct detection of electroactive coproduct of enzymatic reaction. The scientific success led to the practical application—the first glucose analyzer for whole blood was commercialized (Yellow Springs Instrument 23 YSI, 1975). This was the first generation of enzyme-based biosensors where the amperometric signal was related to a product of the enzyme reaction (i.e., H_2_O_2_), but the enzyme active center did not communicate directly or in a mediated pathway with an electrode. In other words, this was an indirect measurement of the enzyme’s substrate (i.e., glucose) following the reaction coproduct formation. Despite the first success, the indirect measurement had many disadvantages, particularly because of interference with easily oxidizable species present in real biomedical liquids (e.g., vitamin C, paracetamol, etc.).

**Figure 22:**
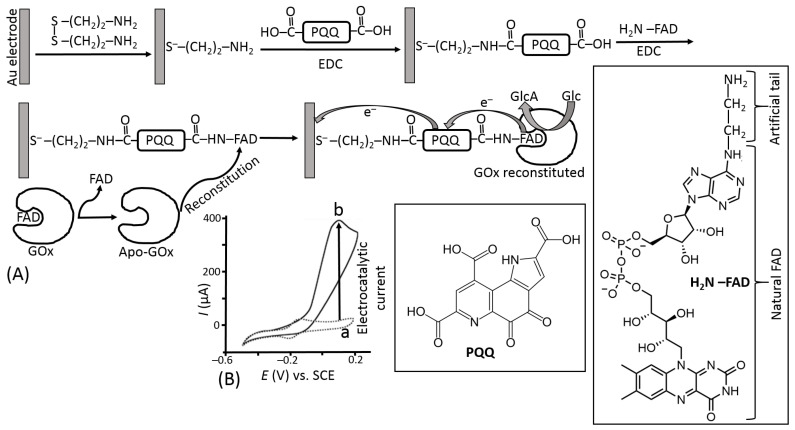
(**A**) Reconstitution of GOx-apo-enzyme (enzyme with the removed active center) on a monolayer composed of an artificial analog of the FAD active centers. PQQ located between the active centers and the electrode surface operates as an intermediate electron-transfer “station” facilitating the electron transport to the electrode surface. (**B**) The cyclic voltammograms show the background current in the absence of glucose (a) and in the presence of glucose, 80 mM (b), potential scan rate, 5 mV s^−1^. Abbreviations used: FAD—flavin adenine dinucleotide, PQQ—pyrroloquinoline quinone, EDC—1-ethyl-3-(3-dimethylaminopropyl)carbodiimide (reagent used in the covalent immobilization process).

The use of electron-transfer mediators shuttling electrons between the enzyme active centers and electrodes allowed the biosensor operation with much fewer positive potentials applied, thus reducing the effect of highly oxidizable interferants (Figure 19a). However, another problem appeared for the second generation of biosensors based on the mediated electron transfer, particularly using the glucose oxidase. Their operation required a much faster reaction of the mediator with the reduced active enzyme center compared with its reaction with O_2_—a native electron acceptor. This was not easy to achieve; thus, many enzyme systems, especially based on GOx, operated only under anaerobic conditions to avoid the H_2_O_2_ formation, which competed with the mediated electron transfer. In order to facilitate the mediated electron transfer and exclude the reaction with O_2_, the mediator should operate with the enzyme in an extremely efficient mode. This can be achieved by different means, mostly increasing the local concentration of the mediator sites near the enzyme molecules in a thin film at the electrode surface. The most efficient mediator enzyme configuration has been reported for GOx molecules with extracted native active center and then reconstituted on its artificial analog attached to an electrode surface [89,90]. This enzyme configuration aligned at the electrode surface allowed fast vectorial electron transfer to the electrode, effectively competing with O_2_ reduction (Figure 22).

The system shown schematically in Figure 22 represents the most efficient, but very sophisticated and difficult, realization example of the mediated electron transfer. Indeed, the assembly of the reconstituted GOx on a self-assembled electron-mediator monolayer is very interesting for demonstrating the electron-transfer efficiency limit (in terms of the electron transfer rate), but impractical for its real use. Many other less sophisticated and practically useful enzyme assemblies with electron-transfer mediators have been reported over decades (see for example the system shown schematically in Figure 21). The great majority of them were based on oxidase enzymes and particularly using GOx. Multistep biocatalytic cascades involving the operation of several enzymes in a sequence can be used for the biosensing of various analytes serving as substrates for different enzymes [91,92,93].

**Figure 23:**
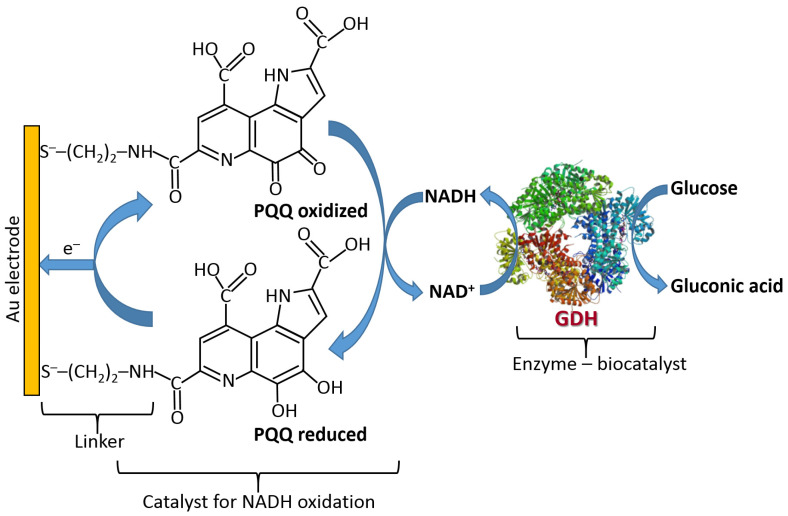
Amperometric glucose analysis with the use of NAD^+^-glucose dehydrogenase (GDH) and PQQ catalyst for recycling NADH back to NAD^+^. Note that this enzyme system is O_2_-insensitive, thus being different from systems based on O_2_-dependent glucose oxidase (GOx). Different catalysts, mostly represented by quinones, can be used to facilitate the electron transfer between NAD^+^/NADH-dependent enzymes and electrodes. The PQQ catalyst shown here is only an example.

NAD^+^/NADH-dependent dehydrogenases require special catalysts for recycling the NAD^+^/NADH cofactors [94] (Figure 23). It should be noted that these catalysts can only facilitate the oxidation of NADH converting it back to NAD^+^; thus, they can operate only in the oxidative pathways with the enzymes. The electrochemical reduction of NAD^+^ is very difficult due to the requirement of two-electron-transfer simultaneously; thus, the direct reductive enzyme processes involving NAD^+^ are not possible in the electrochemical systems.

### 6.4. Biosensors Based on Cells and Cellular Fragments

The previous discussion was mostly concentrated on the use of purified enzymes as biorecognition/biocatalytic units in various biosensors. However, for some specific biosensor applications, a complicated procedure of separation and purification of the enzymes is not needed, and the whole biocatalytic processes performed in intact biological cells can be used. The whole cells (usually microbial) or cellular fragments can be associated with a transducer interface to yield a response to specific biological substrates or inhibitors, and then transformed into an electronic output signal [95,96,97,98]. Microbial cell-based biosensors have been reported for the analysis of a broad variety of biological substrates ranging from small inorganic substances (e.g., CO_2_ [99]) to biorelated molecules (e.g., vitamin B6 [100]). The complexity of biological processes performed in the cells allows various substances to be included in the biocatalytic pathways, then altering the biocatalytic cascades and resulting in variation of measurable concentrations of biomolecules selected as biosensing reporters. In the easiest way, local changes in O_2_ concentration can be used for the generation of an amperometric output signal [101]. This signal corresponds to the change of cellular respiration upon varying biocatalytic pathways in the presence of analyte species. The analysis based on the O_2_ measurements can be considered as indirect biosensing similar to the performance of the first biosensor generation pioneered by Clark. In a more sophisticated approach, the electrons can be shuttled between components of the cellular biochemical cascades and an electrode surface with a mediator’s support [102]. This might be compared with the performance of the second generation of biosensors based on the mediated electron transport. However, in the case of microbial cells, the limitations for selecting the mediator molecules are much stronger than for separated enzymes. Indeed, the mediator molecules should cross the membrane barrier in the cells for transporting charges between an internal part of the cells and external electrodes. In the most efficient and sophisticated systems, the biological membranes can be modified to allow easy charge transport through the membrane itself [103,104].

**Figure 24:**
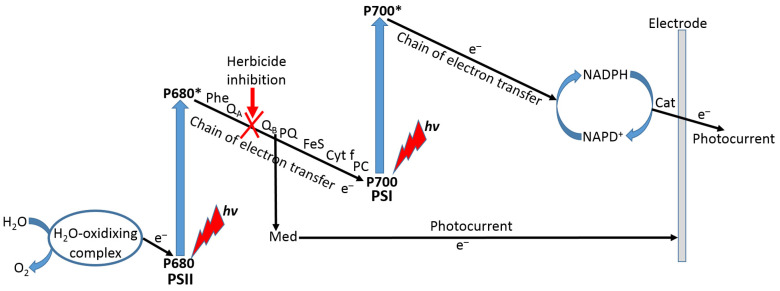
Schematic of photosynthetic electron transport chain (PET) consisting of photosystem II (PSII) oxidizing H_2_O and producing O_2_, and photosystem I (PSI) reducing NADP^+^ to NADPH. P680 and P700 are special pairs composed of chlorophyll molecules being excited with light (P680* and P700* correspond to their excited states). Electron-transfer chain components are abbreviated. The inhibition center and mediated effect can be localized approximately in the middle of the electron-transfer chain between PSII and PSI.

Among many different cellular biosensors developed in recent decades, a special kind designed for biosensing of herbicides can be based on the use of cyanobacteria [105,106] or plant thylakoid membranes [107,108] with inhibition of the photosynthetic electron transport (PET) pathway. In most of the systems based on thylakoid membranes, herbicides act as inhibitors of the PET between the two photosystems (PSI and PSII), then decreasing photocurrent measured by the thylakoid membrane-modified electrode (Figure 24). The photocurrent used as the biosensing reporter signal can be mediated or nonmediated depending on the exact preparation of the modified electrode. In the case of the mediator use, its redox potential can be tuned to inhibit the PET at different positions in the electron transport chain.

### 6.5. Potentiometric Biosensors

Sometimes realization of amperometric biosensing mode might be difficult and impractical. In this case another electrochemical signal transduction mode, such as potentiometric, might be a good solution.

**Figure 25:**
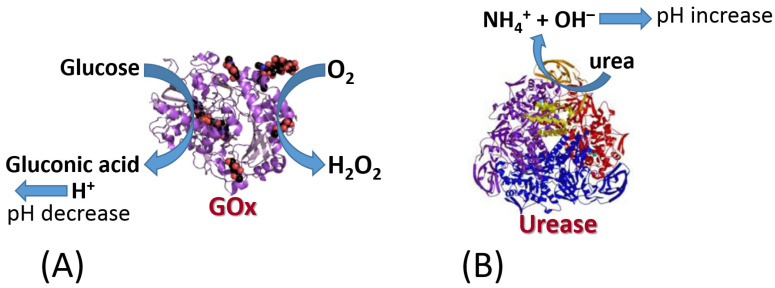
Examples of the pH decrease (**A**) and increase (**B**) produced by enzyme reactions. Notably, in practical applications, the enzymes should be immobilized onto a pH-sensitive transducer surface.

The challenge in the potentiometric biosensing is the specific response to one kind of analyzed species. While the nonbiological approach has been thoroughly developed using various ion-selective electrodes [109] and membranes [110], the biosensing potentiometry is usually based on pH changes produced by enzyme reactions [111,112] (Figure 25). It should be noted that the final pH change generated in the course of a biocatalytic reaction depends on the whole composition of the analyte solution, particularly controlled by a buffer used and its concentration. Assuming for simplicity that buffering equilibrium does not contribute to the pH changes, one can consider that the [H^+^] concentration changes (expressed as the pH changes) are directly related to the enzyme substrate-analyte concentration. In the case of the immobilized enzyme, the pH changes proceed locally (interfacially) and may not propagate to the whole bulk solution [112]. Then, a H^+^ concentration gradient appears, and the local pH change results from the competition of two processes: (*i*) H^+^ consumption (or release) in the enzyme reaction and (*ii*) H^+^ diffusion compensating the biocatalytic process. This significantly complicates the theoretical model, which will be even more complex when the buffer contributes to the resulting pH changes. The pH changes may be nonlinear vs. the analyte concentration and can be affected by the enzyme reaction kinetics, then resulting in a more sophisticated process compared with the direct ion measurements performed with ion-selective electrodes. Additional complications originate from the buffer properties of the enzymes utilized in the biosensing process. Indeed, the protein backbone includes dissociated/protonated amino acid residues contributing to the buffering effect. Notably, the high local concentration of the enzyme in the sensing layer makes this effect significant. Therefore, even in the case when the external buffer effect might be minimized by performing reactions in nonbuffered solutions, the internal buffer effect of the enzyme itself cannot be avoided.

Another biosensing approach based on potentiometric responses has been realized using field-effect transistors (FETs), particularly used in affinity sensors, e.g., represented with immunosensing systems. The affinity complex formation in the presence of the analyte molecules (e.g., antigens) results in charges produced near the gate surface functionalized with a complementary species (e.g., antibodies). The charge changes at the FET gate can be transduced to the electronic output proportional to the target analyte concentration. The biosensing effect can be amplified with a secondary antibody binding, particularly when the antibody is labeled with an enzyme producing pH changes near the pH-sensing interface.

## 7. Brief Conclusions

In conclusion, we need to repeat that the present paper does not pretend to highlight numerous modern biosensing concepts and specific biosensing system examples. On the other hand, this review, formulated more in an educational rather than a scientific way, intends a very generic summary of the biosensing realization, particularly in electrochemical signal transduction mode. The review leaves aside many other kinds of biosensing, particularly not describing various optical biosensors [3]. It should be noted that even electrochemical biosensors were not fully described; for example, electrochemical biosensors based on impedance measurements [113,114] were not mentioned. One should realize that the modern technology applied to biosensing is a huge research area, and even a short summary of it would require a book rather than a review article, even a long one. Indeed, such books exist [115,116], and many comprehensive review articles are available [117,118]; however, they might be not easy for reading by inexperienced newcomers to the area. Therefore, the present review leaves aside numerous modern examples of biosensors and instead concentrates on general concepts, being addressed to students and newcomer researchers as helpful introductory material.

Particularly, the following subtopics related to biosensors were not considered in the present review, but the interested reader can find information about them in the provided references: scanning electrochemical microscopy (SECM) [119], scanning gel electrochemical microscopy (SGECM) [120], bioanalytical chemistry [121,122], microfluidic electrochemical biosensor platforms [123], electrochemical biosensor designs [124], bioelectrochemical sensing [125], electrochemical bioelectricity [126], etc.

It should be noted that biosensors represent important tools for neuroscience [127]. Many examples of biosensor applications for studies in neuroscience are available [128,129,130]. The present review offers a general overview of biosensors, some of which may be easily adopted for neuroscience applications.

## Data Availability

The data is available from the authors upon request.
